# Pathogenicity and virulence of *Helicobacter pylori*: A paradigm of chronic infection

**DOI:** 10.1080/21505594.2024.2438735

**Published:** 2024-12-26

**Authors:** Marguerite Clyne, Tadhg Ó Cróinín

**Affiliations:** a School of Medicine, University College Dublin, Dublin, Ireland; b The Conway Institute of Biomolecular and Biomedical Science, University College Dublin, Dublin, Ireland; c School of Biomolecular and Biomedical Science, University College Dublin, Dublin, Ireland

**Keywords:** *Helicobacter pylori*, virulence, gastritis, duodenal ulcer, gastric cancer, epidemiology

## Abstract

Infection with *Helicobacter pylori* is one of the most common infections of mankind. Infection typically occurs in childhood and persists for the lifetime of the host unless eradicated with antimicrobials. The organism colonizes the stomach and causes gastritis. Most infected individuals are asymptomatic, but infection also causes gastric and duodenal ulceration, and gastric cancer. *H. pylori* possesses an arsenal of virulence factors, including a potent urease enzyme for protection from acid, flagella that mediate motility, an abundance of outer membrane proteins that can mediate attachment, several immunomodulatory proteins, and an ability to adapt to specific conditions in individual human stomachs. The presence of a type 4 secretion system that injects effector molecules into gastric cells and subverts host cell signalling is associated with virulence. In this review we discuss the interplay of *H. pylori* colonization and virulence factors with host and environmental factors to determine disease outcome in infected individuals.

## Introduction

*Helicobacter pylori* is a Gram negative, microaerophilic bacteria, that was only cultured for the first time in 1984 [1], but we now know it has evolved with humans over thousands of years, and is uniquely adapted to live, and cause chronic infection in the hostile environment of the human stomach. In this review we discuss how *H. pylori* colonizes the stomach, establishes persistent infection, and is responsible for a range of disease outcomes in a subset of infected individuals.

### *H. pylori* disease association

The natural habitat of *H. pylori* is the gastric mucosa of humans and non-human primates. Infection results in a complex inflammatory response and the development of chronic antral gastritis, which persists for the lifetime of the host unless the infection is treated with antimicrobials. Despite the gastritis most infected individuals are asymptomatic, but 10–15% of infected people develop peptic ulcer disease, and *H. pylori* is responsible for most duodenal ulcer cases in humans. Early studies in children [2,3] provided strong evidence of the link between *H. pylori* infection in humans, and the development of primary gastritis and duodenal ulceration. Successful eradication of the infection with antimicrobials results in healing of the ulcer, and recurrence is associated with treatment failure or reinfection [4,5]. How *H. pylori* infection of the stomach results in duodenal ulceration was puzzling at first, but studies later showed that in both adults and children the presence of gastric metaplasia and *H. pylori* infection greatly increases the chances of developing duodenitis and duodenal ulceration [6,7]. *H. pylori* can associate with gastric metaplastic cells in the duodenum, and this results in duodenitis and erosion of epithelial integrity resulting in ulceration. Hypergastrinemia and increased production of acid also play a role in development of duodenal ulceration. Increased acid output and fasting and meal-stimulated gastrin production are found in *H. pylori* infected individuals with duodenal ulceration compared to uninfected persons [8,9].

Chronic infection with *H. pylori* is a significant risk factor for the development of gastric cancer with 1% of infected individuals developing the disease, and *H. pylori* is classed as a group one carcinogen by the WHO. Infection induces a series of consecutive progressive changes in the stomach that result in gastric cancer. Upon infection non-atrophic gastritis (NAG) develops, leading to multifocal atrophic gastritis, intestinal metaplasia, and eventually dysplastic changes occur leading to invasive adenocarcinoma. Eradication of *H. pylori* infection can result in regression of precancerous lesions [10,11]. A study in South Korea of first-degree relatives of people with gastric cancer showed that treatment for *H. pylori* reduced the risk of developing gastric cancer [12]. However, it is thought that eradication of *H. pylori* to prevent development of gastric cancer is likely to be most effective if targeted in a young population for whom precancerous dysplastic changes have not yet occurred [13]. In addition, a longitudinal cohort study of over four thousand asymptomatic individuals from Japan and Singapore, indicated that along with *H. pylori* status, consideration of environmental, lifestyle and epigenetic information would greatly increase the efficacy of strategies to prevent gastric cancer development [14].

The development of gastric mucosa associated lymphoid tissue (MALT) lymphoma following *H. pylori* infection occurs in less than 1% of infected people. In a prospective nested case control study with over 230,000 participants from the USA and Norway, the ODDs ratio for the risk of developing gastric MALT lymphoma was 2.8-fold higher in *H. pylori* positive individuals compared to *H. pylori* negative individuals [15]. Recognition of *H. pylori* by tumour-infiltrating T-cells drives B cell proliferation within gastric MALT lymphomas [16,17]. Regression of the lymphoma occurs in up to 80% of patients upon eradication of the infection [18,19].

Infection with *H. pylori* has been shown to correlate with an increased risk of developing colorectal cancer (CRC) [20,21]. However, the human stomach is the only reliable reservoir of *H. pylori* infection. *H. pylori* antigens can be detected in stool specimens and indeed a stool antigen test is used reliably to diagnose infection [22]. However, culture of the organism from normal stool specimens has been reported on only a handful of occasions [23,24], suggesting culture is difficult, likely due to low numbers or absence of viable organisms in faecal samples. A recent study used data from four large European genetic data pools, and a Mendelian randomization approach to assess if the presence of single nucleotide polymorphisms (SNPs) associated with *H. pylori* infection could show possible causality between *H. pylori* and CRC, but no association was found [25]. Recently a potential mechanism whereby infection with *H. pylori* could result in the development of CRC was identified [26,27]. Infection of APC mutant mice (mice prone to intestinal and colonic tumour development), with *H. pylori* caused accelerated intestinal and colonic tumour development. This phenotype could be reversed by eradication of infection and was not seen in germ free mice, indicating a role for the microbiome. Infection resulted in a reduction in regulatory and proinflammatory T cells, induction of pro-carcinogenic STAT-3 signalling in the epithelium, and a decrease in goblet cells. Examination of colonic biopsy specimens from *H. pylori* infected patients with CRC showed similar epithelial and immune cell alterations. Infection of wild type mice resulted in increased abundance of the mucin degrading *Akkermansia* and *Ruminococcus* species. It was proposed that *H. pylori* infection results in disruption of mucosal integrity, and without an adequate regulatory T cell response inflammation occurs resulting in cancer development in the colon. Furthermore, profiling of the virome of *H. pylori* infected APC mice found that infection was associated with a distinct virome, of which over 90% were temperate phages, many of which are thought to be able to infect bacteria associated with colonic and rectal cancer [28]. Metagenomics demonstrated an expansion of these temperate phages in APC mice infected with *H. pylori* at the early stage of CRC and suggested that prophage induction could be triggered by infection resulting in virulent phage gene expression [29]. There is a requirement now for studies with large numbers of clinical samples to determine the relevance of this mechanism in mediating CRC in humans.

### *H. pylori* epidemiology

Over 50% of the global population is estimated to be infected with *H. pylori* [30]. Using data from a systematic review and meta-analysis of over 180 published reports from sixty-two countries, it was estimated that the number of infected people in 2015 was approximately 4.4 billion. Africa had the highest prevalence of infection (70.1%). Among individual countries, there was considerable variation in prevalence, with infection in Switzerland estimated to be as low as 18.9%, and infection in Nigeria to be as high as 87.7% [30]. Infection is higher in low- and middle-income countries than in wealthier countries, and risk factors for acquisition of infection include overcrowding, living in a household with other infected people, and poor standards of hygiene, including lack of access to clean water [31]. Since the discovery of *H. pylori*, the prevalence of infection has declined rapidly in high-income countries, a decline that cannot be explained by treatment strategies alone. Significant improvements in living conditions are thought to have resulted in less opportunity for transmission to occur.

Infection with *H. pylori* typically occurs in early childhood, and lasts for the lifetime of the host unless treated with antimicrobials. Infection usually occurs before the age of three [32–34], and infection or re-infection in older children and adults was shown to be rare in a developed country [34,35]. A significant risk factor for infection is if your mother/primary caregiver and/or older siblings are infected [31,36]. Transmission of infection between spouses is not common [37–40].

Exactly how *H. pylori* is transmitted from person to person is not known. *H. pylori* could be isolated reliably from vomitus of infected persons treated with an emetic, but only occasionally from saliva and cathartic stools [41]. A likely method of transmission is gastric-oral [42] with exposure to a *H. pylori* infected person with gastroenteritis increasing the chances of becoming infected, and possibly oral-oral but colonization of the mouth with *H. pylori* is thought to be transient [43,44]. Faecal-oral transmission is also a possibility [45] but culture of *H. pylori* from stool is very difficult [23,41]. Transmission through contaminated water supplies may play a role but how long *H. pylori* can survive in water outside of a microaerophilic environment is not known. There is evidence that *H. pylori* may survive inside free living amoeba found in water [46] and this might provide *H. pylori* with a favourable environment. Microcolony formation on vegetables can occur [47] and so biofilm formation on food sources or other surfaces could also potentially play a role in enabling survival of *H. pylori* outside the human body and aid in transmission of infection. However, it should be noted that to date, definitive transmission of *H. pylori* infection via food or water has not been proven.

**Figure 1.**
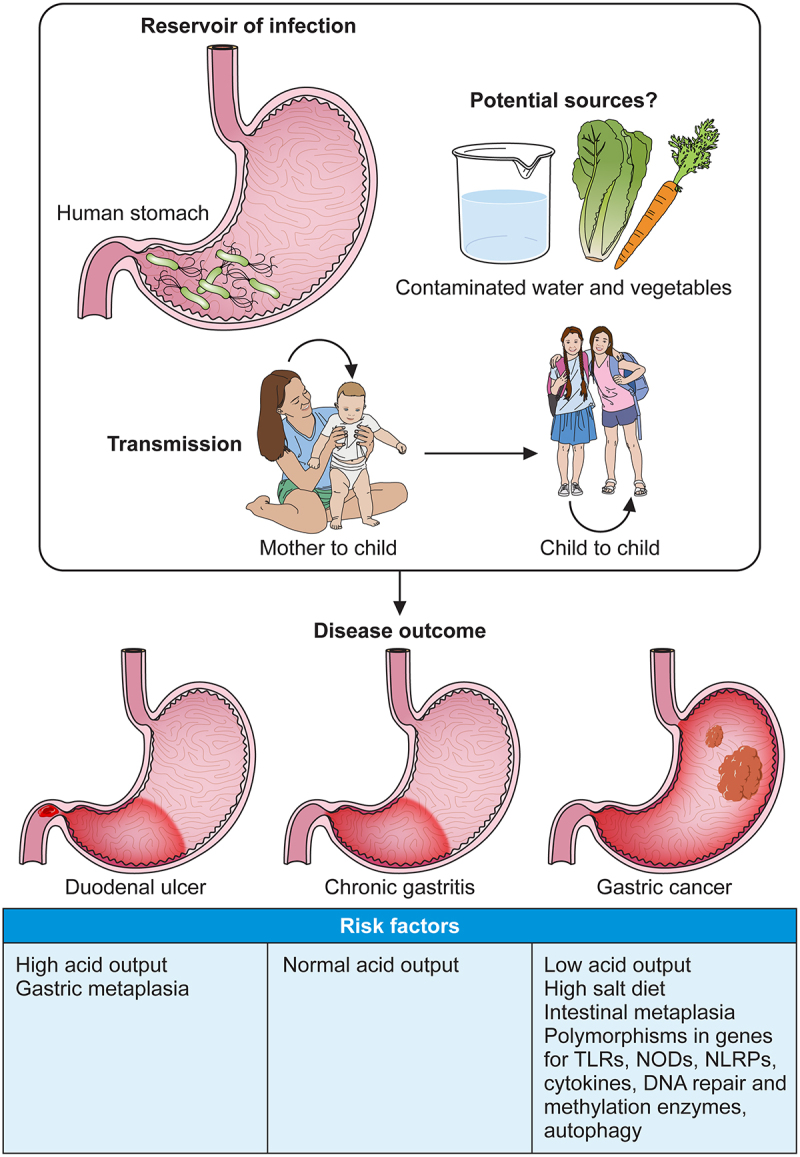
Summary of *H. pylori* transmission routes, disease outcome, and host risk factors.

The outcome of *H. pylori* infection is different for different individuals, but outcome also differs throughout the world for different ethnic groups. Gastric cancer incidence is high in Eastern Asia, Eastern Europe, and Central and South America [48], but the incidence of both gastric cancer and of peptic ulcer disease is relatively low in Africa [49], despite there being a high prevalence of infection on the continent. While bacterial factors play a role in mediating disease outcome, the presence of polymorphisms in host genes, the occurrence of which may vary in different populations, may also play a role. Polymorphisms in genes encoding for Pathogen Recognition Receptors and cytokines have been recognized as host factors that can influence the outcome of infection [50,51]. Polymorphisms in the IL-1β gene cluster and TNFα, both proinflammatory cytokines and inhibitors of gastric acid secretion have been identified as risk factors for the development of gastric cancer [52,53]. Proinflammatory genotypes of TNFα and IL-10 were each associated with increased risk of non-cardia gastric cancer, but not for oesophageal cancer or gastric cardia cancer [53]. The risk of developing gastric cancer is greatly reduced in individuals who have a duodenal ulcer [54]. In a case control study that included over 500 Mexican adult patients with diverse gastroduodenal symptoms, polymorphisms in TLR9 and TLR5 were found to result in significantly less IL-1β and TNFα expression. Polymorphisms in TLR-5 also reduced the levels of cytokines IL-6 and IL-10. However, polymorphisms in TLR5 were not found to be associated with disease, while a polymorphism in TLR9 was found to be associated with an increased risk of developing a duodenal ulcer [55]. Many studies examining the role of host genetic polymorphisms on the outcome of *H. pylori* infection, and in different ethnic groups are confounded by a low number of participants, and lack of appropriate control subjects. A further confounding factor is that cytokine gene polymorphisms have also been associated with infection by more virulent *H. pylori* strains [56]. There is a need for larger studies to be done in different populations which also include consideration of bacterial and environmental factors for more definitive conclusions to be made. Figure 1 summarizes known and potential transmission routes for *H. pylori*, possible disease outcomes, and host risk factors.

### *H. pylori* colonization factors

Examination of biopsy specimens from infected individuals reveals that the majority of *H. pylori* organisms, approximately 80%, are found in the gastric mucus layer, with most of the remaining organisms adherent to surface epithelial cells and in the gastric pits [57]. A much smaller number of organisms colonize deeper in the gastric glands where they are found growing in microcolonies and interacting directly with gastric progenitor and stem cells [58]. Although *H. pylori* is largely an extracellular bacterium, intracellular *H. pylori* have been found in both epithelial and phagocytic cells [59,60]. Survival of *H. pylori* in an intracellular niche could allow it to evade killing by extracellular antibiotics and cells of the immune system. Before the discovery of *H. pylori* it was thought that no microorganisms lived in the stomach. We now know that the stomach does harbour a microbiome, however, when *H. pylori* infects it can become the dominant organism [61]. Despite being a neutralophile it can survive in an acidic environment [62] and possesses an impressive arsenal of factors that enables it to colonize the gastric mucosa and cause chronic infection.

#### Urease

*H. pylori* possesses a very potent urease enzyme that can hydrolyse urea present in the stomach [63] and is essential for colonization [64,65]. Non-*H. pylori Helicobacter* species that infect the stomachs of animals have also been shown to have urease activity [66] underlining the key importance of this enzyme for survival of the species in the stomach. Urease is found both on the bacterial surface and intracellularly in the cytoplasm of the bacteria [67–69]. Urease breaks down urea to generate ammonia and CO_2_ and the ammonia generated contributes to increasing the pH of the local environment, and thus protects the bacteria from the effect of acid. However, raising the pH of the local environment *in vivo* is not enough to enable colonization by urease negative mutants suggesting that intracellular urease activity is needed for survival in the stomach [65]. Intracellular urease controls the pH of the periplasm, thus ensuring protein synthesis can occur even when the extracellular pH is low [69,70]. Urea is taken up by the cell through a proton-gated channel, UreI, which is only active at low pH, and therefore urea cannot be taken up by the bacteria at neutral pH [71]. The conversion of CO_2_ to HCO_3_- by the periplasmic α-carbonic anhydrase enzyme also contributes to maintenance of a neutral periplasmic pH [72]. Infection of 3D gastric organoids, and cells derived from gastric organoids growing in 2D monolayers, has revealed recently that *H. pylori* preferentially attaches to large differentiating gastric pit cells. This attachment is governed by chemotaxis of the organism towards urea released by the cells, the amount of which correlates with the size of the cells [73]. The production of a potent urease by *H. pylori* suggests that in the presence of urea the organism could not survive unless it was in an acidic environment, as production of ammonia at higher pH would increase the local pH above pH 7.0, and *H. pylori* cannot survive in alkaline conditions [62].

Other roles that are attributed to urease of *H. pylori* include reducing mucus viscosity, disruption of epithelial integrity, induction of proinflammatory cytokines, and protection from reactive oxygen species. Urease mediated production of ammonia, and increase in local pH results in the lowering of mucus viscosity, thus facilitating *H. pylori* motility, and penetration of gastric mucus [74]. Infection of MKN28 cells, gastric cancer epithelial cell line, grown on transwell filters, with wild-type *H. pylori* resulted in re-distribution of the tight junction protein occludin, but not when cells were exposed to a *ureB* knockout mutant. This urease-dependent disruption in barrier integrity was mediated via an increase in phosphorylation of myosin II regulatory light chain [75], a known regulator of tight junction formation [76]. Treatment of primary human blood monocytes with purified *H. pylori* urease resulted in a dose-dependent production of proinflammatory cytokines IL-1β, IL-6, IL-8, and TNFα. Cytokine production was inhibited by preincubation of the cells with antibodies against *H. pylori* whole cells, purified urease, or a urease subunit [77]. Recombinant urease could activate mucosal macrophages to produce IL-1β, IL-6 and TNFα but not IL-8 [78]. Stimulation of cultured primary human gastric epithelial cells with purified urease resulted in expression of IL-6 and TNFα but not IL-8 [79].

A non-catalytic function of *H. pylori* urease identified recently is protection from reactive oxygen species (ROS). Exposure of *H. pylori* to epithelial cells or to phagocytes results in the generation of toxic ROS [80], and *H. pylori* expresses catalase and superoxide dismutase for protection. Non catalytic catalase has also been shown to protect against ROS through oxidant quenching of its Met residues via reduction of Met-SO by methionine sulphoxide reductase (Msr) [81]. Urease contains twenty five Met residues. Catalytically inactive urease mutant strains that contained all the Met residues were better able to withstand exposure to HOCI compared to a *ureAB* deletion mutant. Mass spectrometry demonstrated that eleven Met residues in urease were susceptible to oxidation but subsequently could be repaired by Msr [82].

Urease is expressed by *H. pylori* throughout infection, even when the bacteria is found deep in gastric mucus where the pH is close to neutral. Using a tetracycline repressor-based system, that binds to specific operator sequences in the target promoter and silences transcription of the downstream gene, conditional urease knockout mutants were constructed. Mice challenged with these mutants only became infected when anhydrotetracycline (ATc) a potent tetracycline inducer was administered at the time of challenge to remove the repressor from the operator sequence. Two weeks later ATc was withdrawn from the animals to repress urease expression again, and within 5–7 days bacteria could no longer be detected in the stomachs of the animals. This showed that urease expression is necessary not just for establishment of infection, but also for maintenance of infection. Interestingly urease positive *tet-*escape mutants were detected in mice challenged with the conditional mutant in the presence of ATc, and then left without ATc for up to 42 days [83], demonstrating the strong selective pressure on *H. pylori* to restore urease expression for maintenance of chronic infection *in vivo.*

#### Motility

**Figure 2.**
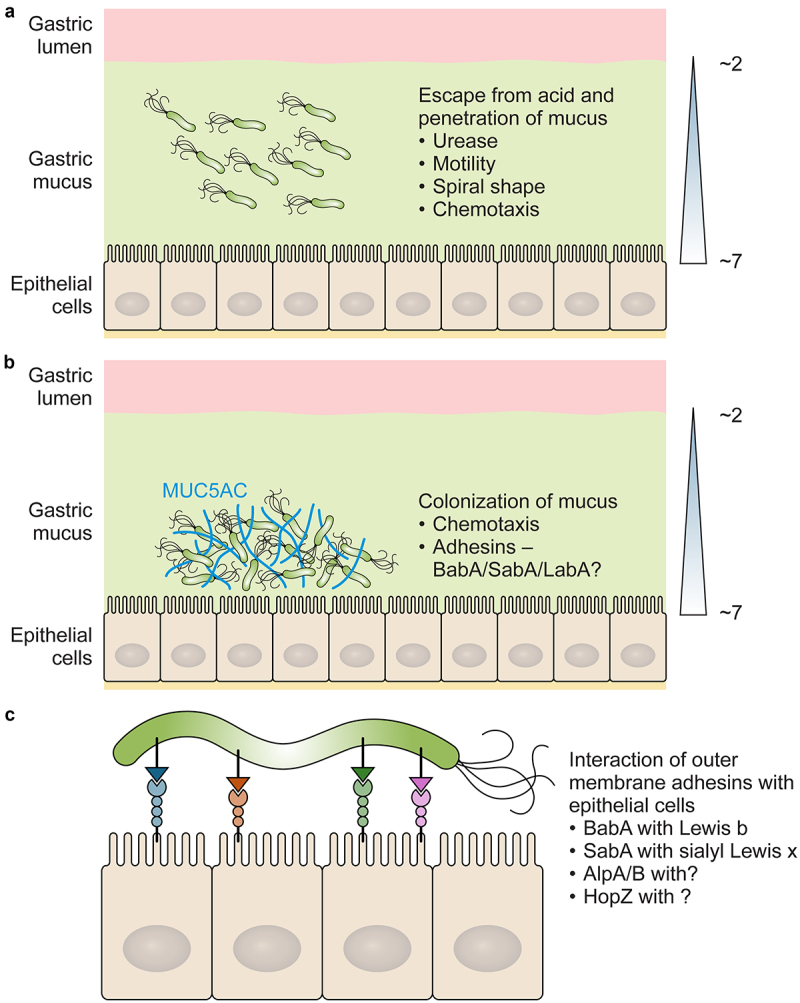
*H. pylori* colonisation of gastric mucus and interaction with epithelial cells. A. *H. pylori* escape from gastric acid. Urease protects *H. pylori* from the effect of gastric acid, the organism quickly traverses gastric mucus to escape the acidic environment in the lumen using flagella mediated motility, chemotaxis, and its spiral shape. B. *H. pylori* colonizes gastric mucus by locating itself close to the epithelium and binding via outer membrane protein adhesins to glycans present on MUC5AC . C. A proportion of bacteria interact with carbohydrate and other unidentified receptors present on the gastric epithelial cells.

Experimental infection *of Helicobacter felis* in mice and *H. pylori* in gerbils found that both organisms localize to a narrow pH gradient in the mucus layer close to the epithelial surface. Disruption of the pH gradient in the mucus layer disrupted the spatial orientation of the bacteria. The organisms were found throughout the mucus layer and were quickly killed when exposed to low lumen pH [84]. Flagellar mediated motility is essential to enable *H. pylori* to reach the epithelium away from the harmful effect of acid (Figure 2a). Motility is conferred on *H. pylori* via 3–6 sheathed polar flagella. The flagella sheath is thought to protect the flagella filament from the effect of gastric acid and may mediate adherence. The lipoprotein HpaA that mediates adherence to gastric cells and can induce TNFα production in macrophages [85] has been reported to localize specifically to the flagellar sheath [86]. However other investigators reported variation in the surface localization of HpaA even within the same population of bacteria. Using a monoclonal HpaA antibody and immunoelectron microscopy HpaA was found concentrated at the poles of the bacterial cell, on the flagella sheath and on the cell wall of the bacteria [87]. Another protein reported to be localized to the flagellar sheath is FaaA (flagellar-associated autotransporter A) [88], a protein secreted by the type V (autotransporter) pathway. However, FaaA has also been found in flagellar negative organisms [89] and its function is not clear.

*H. pylori* flagella was one of the first bacterial factors to be shown to be essential for colonization of experimental animals. Flagellar negative mutants were unable to colonize gnotobiotic piglets, and both flagellin proteins FlaA and FlaB were necessary for persistent infection [90]. Later work demonstrated that it is the motility conferred by flagella, rather than the presence of flagella alone, which enables *H. pylori* to establish infection [91]. Flagellar biogenesis is stringently regulated. Deletion of FlbA, the regulator which controls expression of *flaA, flaB* and *flgE*, results in lack of motility [92], and a decrease in colonisation rates [93]. The quorum sensing auto-inducer 2 (AI-2) protein can regulate expression of flagellar genes [94,95] and mutants deficient in LuxS have reduced motility and infectivity rates compared to the wild-type parental strain [96], suggesting that *H. pylori* may regulate expression of its flagellar genes in response to bacterial density.

*H. pylori* flagellin filaments are decorated with a pseudaminic acid (PSE) sugar derivative, similar to sialic acid found on mammalian cells, which appears to be essential for motility and construction of flagella filaments [97]. *H. pylori* mutants deficient in a deglycosylase, HP0518, had increased levels of PSE on FlaA, were hypermotile, and associated more closely with gastric epithelial cells inducing activation of NF-κB. Infection of C57/BL/6 mice resulted in higher numbers of mutant bacteria associated with gastric tissue compared to infection with wild type organisms [98]. Thus, the levels of flagellin glycosylation seem to regulate motility and the interaction of *H. pylori* with epithelial cells.

Bacterial flagellins can normally activate TLR5, part of the host innate immune system. However, *H. pylori* flagellins are unable to activate TLR5 [99,100]. This is because *H. pylori*, in common with other flagellated organisms that are unable to activate TLR5, have evolved to alter the amino acids found in the D1 N terminal domain of the TLR5 recognition sequence [101]. This lack of recognition by TLR5 is a mechanism whereby *H. pylori* can evade the host immune response and establish persistent infection. Interestingly it has been shown recently that *H. pylori* can activate TLR5 but in a flagellin independent manner [102,103]. This is discussed in more detail below.

#### Chemotaxis

*H. pylori* possesses a sophisticated chemotaxis system which enables it to sense its environment, locate nutrients and the presence of urea, detect pH and ROS, and find a favourable niche in the human stomach. The chemotaxis signal transduction system in *H. pylori* contains core chemotaxis proteins, including the chemoreceptors, TlpA-D, CheW coupling protein, CheA kinase, and CheY response regulator. Auxiliary proteins include CheV1–3, CheZ phosphatase, and a unique chemotaxis protein, ChePep [104]. ChePep plays a role in regulation of flagellar rotation, critical for chemotactic behaviour and mutants lacking ChePep are outcompeted by WT bacteria and are unable to colonize gastric glands in mice [105]. Environmental signals detected by the chemoreceptors are relayed to CheA via CheW or CheV1 [104]. Mutants lacking CheW, CheA, and CheY are all non-chemotactic. Non-chemotactic mutants do not colonise mice efficiently [106], do not induce as much inflammation as WT bacteria [107], and they are found in the murine stomach corpus, but not in the antrum, demonstrating the importance of chemotaxis for colonization in all regions of the stomach [108].

TlpD driven chemotaxis has been shown to control proliferation of *H. pylori* in the murine stomach antrum, but not in the corpus where inflammation is less [109]. Motility and chemotaxis have been shown to be essential for bacterial accumulation at sites of gastric injury. The key role of TlpB in this process was demonstrated when *tlpB* mutants were unable to preferentially accumulate at damaged sites in cultured murine gastric organoids [110]. TlpB was also shown to be the main chemoreceptor responsible for chemotaxis of *H. pylori* towards metabolites produced by human gastric organoids or polarized epithelial cells. Urea was identified as the main metabolite responsible for the chemotaxis and *tlpB* mutants could not establish persistent infection in mice [111].

Using videomicroscopy and a chemotaxis microgradient assay both TlpA and TlpD were identified as independent acid sensors. Approximately 100-fold more mutant bacteria lacking both *tlpA* and *tlpD* were required to establish infection compared to the wild type strain [112]. Disruption of *tlpA* by insertion of a *cat* cassette resulted in loss of chemotaxis towards the amino acid arginine and bicarbonate ions [113]. Arginine is an essential amino acid for *H. pylori* and a substrate in the urea cycle, a metabolic pathway involved in *H. pylori* nitrogen metabolism. Bicarbonate is secreted by epithelial cells in the stomach and plays a role in local pH neutralisation. Ligand binding arrays demonstrated that TlpA can bind several ligands, including the chemoattractants arginine, fumarate, and cysteine. Another high-affinity ligand identified was glucosamine which acted as an antagonist able to block chemoattractant responses, and binding of chemoattractant ligands [114]. TlpC has been shown to mediate the chemoattractant response towards lactate [115], the uptake of which has been shown recently to play a role in the generation of bacteria resistant to complement [116]. TlpD of *H. pylori* is the only cytoplasmic chemoreceptor protein and has been shown to be essential for persistent colonisation in the gerbil model of infection [117]. Studies using WT and *tlpD* mutant bacteria to infect WT mice and mice defective in production of either epithelial cell generated ROS or immune cell generated ROS, suggested that TlpD acts to sense ROS which is a chemorepellent for *H. pylori*, and that the sensing of ROS plays a role in governance of gastric gland colonization [118]. In contrast to these findings, a study using physiological concentrations of HOCl, and an assay using an *in vitro* reconstituted chemotaxis signalling complex, indicates that *H. pylori* is attracted to HOCl which acts to reversibly oxidize a cysteine residue in the binding motif of TlpD, and inactivate the chemotransduction signalling complex. *H. pylori* was found to be resistant to mM concentrations of HOCl [119], a strategy which would aid in enabling persistent infection in inflamed gastric tissue infiltrated by HOCl producing immune cells.

#### Spiral shape

The distinct helical shape of *Helicobacters* has been shown to confer a colonization advantage on *H. pylori*. Seven genes (*csd1–7*) that work to enable changes in cell shape, including functioning as peptidases that hydrolyse peptide cross links in *H. pylori* cell wall peptidoglycan have been identified [120–123]. Up to 28 genes that play a role in altering the morphology of *H. pylori* were identified by flow cytometry profiling of a *H. pylori* deletion mutant library to detect changes in light scattering caused by cell shape changes [122]. Cell shape and motility were studied using optical and electron microscopy and live cell imaging was used to examine the swimming behaviour of bacteria in viscous solutions containing various polymers. Results indicated that alteration in cell shape and in number of flagella promote motility in viscous environments such as gastric mucin [124]. Colonization studies in mice with *csd* gene deletion mutants with curved cell shape were out-competed by WT helical bacteria [121]. Furthermore, studies in mice with WT bacteria and a *csd6* deletion mutant with a straight rod morphology showed that the mutant bacteria were unable to colonize antral glands efficiently, and although they could colonize, proliferate, and persist in corpus glands, inflammation and disease progression was much reduced [124]. Thus, helical shape plays a role in penetration of gastric mucus, establishing infection in niche locations and inducing inflammation.

### *H. pylori* colonization of gastric mucus and adherence to epithelial cells

Once located in the mucus layer in the stomach *H. pylori* needs a mechanism to avoid being removed by mucus flow and epithelial cell turnover. The mucin MUC5AC is produced and secreted by the surface gastric epithelial cells and the deeper gland mucus-secretory cells produce the mucin MUC6 [125]. The gastric adherent mucus gel layer is formed by overlapping layers of MUC5AC and MUC6 mucins [126] and *H. pylori* is preferentially found associated with MUC5AC [126–128]. Members of the *H. pylori* outer membrane family of proteins, referred to as the HOP family, interact with gastric mucin O-linked oligosaccharides and carbohydrate and protein receptors expressed on epithelial cells. The importance of the interaction of *H. pylori* with host structures is underlined by the large number of outer membrane proteins encoded for by its genome [129], many of which have been identified as adhesins. The most well characterized of these adhesins are the difucosylated Lewis b blood group antigen, and the sialyl Lewis x binding adhesins BabA and SabA respectively. The interaction of *H. pylori* with gastric mucins and epithelial cells to establish colonization of the stomach is summarized in Figure 2.

#### BabA

The 78 kDa outer membrane protein adhesin BabA mediates binding to the fucosylated H-1 and the Lewis b blood group antigens found on gastric mucin and on the epithelial surface and is encoded for by the *babA2* gene [130]. Another gene, *babA1* is silent and has an incomplete signal peptide. A *babB* gene is highly homologous to *babA*, except for a central region that determines Lewis b binding specificity. Silent *babA* sequences can be activated by recombination into the *babB* locus resulting in expression of a BabB/A chimeric protein that can bind to Lewis b but is expressed at a low level and is subject to frameshift phase variation [131]. Sequencing of clinical isolates and isolates recovered after experimental infection of Rhesus Macque monkeys showed that the *babA* gene could be deleted or replaced by *babB*, or not expressed due to alteration of dinucleotide CT repeats in the 5’ coding region [132]. Similar results were found following *H. pylori* challenge of mice and gerbils [133]. It has been suggested that there is selective pressure in Rhesus Macques for loss of *babA* and overexpression of *babB*, and that this may confer a fitness advantage on *H. pylori* with modification of BabA expression during infection allowing for adaptation of the organism to inflammation, and alteration of receptor expression at the epithelial surface [134]. BabA expression is also responsive to the effect of pH with a reduction in binding occurring at low pH that can be restored by raising the pH [135]. This reversible pH mediated binding may facilitate the escape of *H. pylori* from mucus or epithelial cells that are released into the gastric lumen with normal mucus and cell turnover. In support of this, in the same study, profiling of clinical isolates and isolates from experimentally infected Rhesus Macques that developed either gastritis or gastric cancer, revealed *H. pylori* can evolve through mutation and recombination of *babA* genes to adapt to different pH conditions in the stomach. Isolates from the corpus, where the mucus is thinnest displayed more acid sensitive binding than isolates from the antrum where a thick protective layer of mucus is present [135]. Furthermore, *babA* mediated binding of *H. pylori* to carbohydrate receptors is dictated by the expression of blood group antigens in the human stomach. Isolates from populations that express A, B and O antigens demonstrate binding to Lewis b, ALewis b, and BLewis b, while individuals from populations that predominantly express blood group O demonstrate majority binding only to H-1 and Lewis b antigens [136]. It is thought that repeated cycles of selection for binding and non-binding activity would only allow for selection of Lewis b binding in individuals with blood group O, and *babA* alleles mediating binding to blood group antigens in the host have arisen via mutation and recombination events. The dynamic nature of BabA mediated binding of *H. pylori* to blood group antigens allows for *H. pylori* to cause chronic infection and persist in the human stomach despite the presence of inflammation and alterations in acid secretion.

#### SabA

The 66 kDa outer membrane protein SabA mediates binding of *H. pylori* to sialylated Lewis x and Lewis a. Upon infection the inflammation induced leads to increased expression of sialylated structures which SabA can bind to [137] thus, enabling persistent chronic infection to occur. It has also been suggested that SabA may play a major role in mediating initial attachment of the bacteria to uninflamed gastric tissue in individuals who do not express Lewis b on gastric tissue [138]. SabA can interact with N-acetyllactosamine-based gangliosides with terminal α3-linked NeuAc [139]. Two minor components of acid glycosphingolipids from human stomach were also identified using TLC separation of glycosphingolipids and overlay assays with WT *H. pylori* strain J99 and a SabA deletion mutant. These were subsequently identified using mass spectrometry, monoclonal antibody staining, bacterial binding assays and inhibition with specific lectins, as Neu5Acα3-neolactohexaosylceramide and Neu5Acα3-neolactooctaosylceramide [140]. Characterization of the three-dimensional structure of the extracellular adhesion domain of SabA and surface plasmon resonance binding assays demonstrated that this domain determines sialyl Lewis x binding specificity and binds, albeit with greatly reduced affinity, to Lewis x [141]. Like BabA, SabA mediated binding exhibits on/off phase variation [137] and multiple alleles of *sabA* can exist in a single *H. pylori* population *in vivo* [142].

*H. pylori* infection is characterized by generation of an inflammatory reaction and rapid recruitment of neutrophils to the site of infection. SabA binding to sialylated receptors on human neutrophils plays a key role in the activation of neutrophils following infection, uptake of the bacteria by phagocytosis, and induction of the oxidative burst, with SabA mutants unable to adhere to the cells or activate them [143]. SabA interacts with gangliosides on red blood cells with the NeuAcα2-3 Gal-disaccharide being the minimal epitope required for binding. Binding to red blood cells is responsible for the sialic acid dependent haemagglutination activity observed with *H. pylori*. Polymorphism in SabA binding to sialylated structures occurs. Five different binding patterns were seen when isolates were tested for binding to sialylated diLewis x, sialylated Lewis a and sialylated N-acetyllactosamine (Ln) glycans. Differences in binding affinities for the tested glycans related to complexities in fucosylation, and to the type of lacto series core chains. Preferential binding to the sialylated diLewis x was the most common binding mode among clinical isolates [144].

Transcription of *sabA* is under the control of the two-component acid responsive signal transduction system ArsRS, and deletion of the histidine kinase locus *arsS* resulted in enhanced binding to gastric epithelial cells in strains with an in-frame *sabA* locus [142]. DNA binding assays, and *in vitro* transcription assays demonstrated that repression of *sabA* transcription occurs by binding of phosphorylated ArsR to DNA, and this binding is dependent on DNA topology [145]. Expression of *sabA* is downregulated in the presence of lactate, and this was dependent on the two-component ArsRS system [146]. A high salt diet and infection with *H. pylori* both promote gastric cancer development in experimental animals [147] and in humans [148,149]. RNA Seq and RT-PCR analysis of *H. pylori* grown in the presence of a high salt concentration demonstrated that *sabA* expression along with another outer membrane protein adhesin gene, *hopQ* (discussed below), was upregulated [150]. Thus, we see that environmental conditions can play an important role in modulation of adherence mediated by SabA. Studies have also suggested that SabA plays a role in promoting *H. pylori* colonization of metaplastic cells. Treatment of mice with tamoxifen induces expression of SPEM (spasmolytic polypeptide expressing metaplasia), which is a step towards gastric atrophy. There is increased expression of sialyl Lewis x in SPEM tissue glands. Exposure of agarose embedded free floating 100 µm thick sections of murine SPEM tissue to *H. pylori* resulted in bacterial adherence deep in the glands, and this binding could be inhibited with 3’sialyl lactose suggesting a role for SabA [151]. Interestingly a recent study using the *Mist1-Kras* mouse model, which has increased expression of metaplastic tissue, has indicated that the SabA paralogue, SabB, promotes colonization of both healthy and metaplastic tissue, but is especially important for colonization of metaplastic tissue glands [152]. This study further underlines the importance of modulation of adhesin expression in *H. pylori* to enable it to adapt to and persist in different environments that arise because of infection.

#### LabA

Another member of the Hop family is the 77 kDa protein LabA. LabA was identified by a competitive exclusion mucin binding assay using LacdiNac, a glycan shown to be specifically expressed on gastric foveolar epithelial cells, matching MUC5AC expression and localization of *H. pylori*. Treatment of either bacteria with LacdiNAc glycoconjugate or LacdiNac purified from human mucin, or treatment of gastric tissue sections with antibodies against LacdiNac resulted in up to a 60% reduction in binding of LabA expressing and non-BabA/SabA expressing strain 26695 to gastric biopsy tissue [153]. However, ligand binding studies with recombinant LabA did not show binding to LacdiNAc in an ELISA type assay, and native electrospray ionization mass spectrometry showed only a weak affinity [154]. Likewise, LabA expressing strains of *H. pylori* did not bind to engineered recombinant mucins carrying the LacdiNAc determinant on different mucin core chains [155]. Together these results suggest that the receptor for LabA might not be LacdiNAc. Further studies are needed to characterize the role of this protein in mediating *H. pylori* adherence to and colonization of the gastric mucosa.

Like *sabA*, *labA* expression has also been shown to be regulated by the ArsRS two component system. Significant increases in the transcript levels of both *sabA, labA* and another outer membrane protein gene, *hopZ*, were seen in an *arsS* mutant, and ArsR bound to the promoter region of each gene [156]. Also, in common with *sabA*, *labA* expression is downregulated in the presence of lactate. However, *labA* downregulation in the presence of lactate was not dependent on the ArsRS system, but on the two component CheA/CheY system [146] required for *H. pylori* chemotaxis to gastric mucin, and for efficient colonization of experimental mice [106]. Interestingly, *H. pylori* has been shown recently to use uptake of host L-lactate to generate bacteria that are resistant to killing by the classical complement pathway. In the presence of lactate accumulation of the complement component C4b on the bacterial surface is blocked. Mutants that are unable to take up lactate are sensitive to killing by complement and are deficient in colonization of experimental mice [116]. It would be intriguing to investigate if lactate mediated changes in outer membrane protein expression plays a role in inhibition of C4b interaction with the bacteria. Figures 2b,c summarize the interaction of *H. pylori* with mucins and gastric epithelial cells.

#### AlpA and AlpB

Screening of a *H. pylori* transposon mutant library identified two independent mutant strains exhibiting a severe reduction in binding to gastric epithelial cells and gastric tissue [157,158]. Two genes, *alpA* and *alpB*, encoding for outer membrane proteins AlpA and AlpB respectively, have since been shown to be required for efficient colonization of mice and gerbils [159,160]. Sequence differences have been observed in the *alpAB* loci of East Asian and Western strains of *H. pylori*. Furthermore, deletion of *alpAB* in East Asian strains reduced *H. pylori* induced IL-8 expression by gastric epithelial cells, and activation of Jun N-terminal kinase, c-Jun, and NFkB occurred only with *alpAB* East Asian strains [161]. The only receptor identified to date for AlpA and AlpB is the basement membrane protein laminin [162].

#### HopZ

Another outer membrane protein that has been identified as an adhesin mediating binding to gastric epithelial cells is HopZ. Two alleles of the *hopZ* gene exist, and expression is regulated by slipped-strand mispairing within a CT dinucleotide repeat motif in the signal-peptide coding region. Expression only occurs in those strains with an open reading frame encoding for a complete protein [163]. In a human volunteer study, *H. pylori* with a functional *hopZ* was isolated from 24 out of 33 individuals challenged with a non-functional or *hopZ* off strain. However, collection of sequential isolates from 26 infected individuals with chronic infection showed no change in *hopZ* status, indicating that *hopZ* status selected for in early infection is stable [164]. To date no receptor has been identified for HopZ.

### *H. pylori* virulence

**Figure 3.**
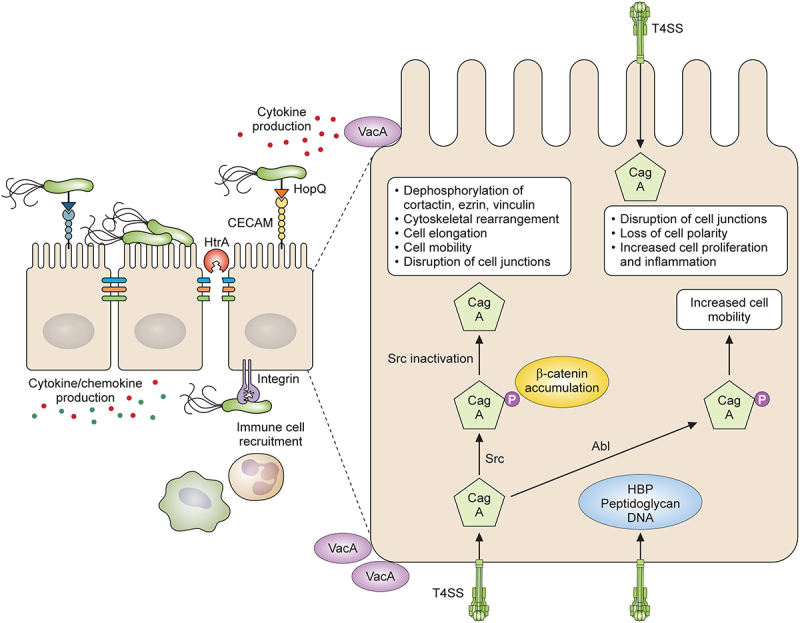
*H. pylori* interaction with gastric epithelial cells and CagA and VacA virulence factors. *H*. *pylori* interacts with epithelial cells via the interaction of outer membrane adhesins with receptors present on epithelial cells. HopQ interacts with CECAM receptors on the cell and the bacteria express the *cag*T4SS enabling effector molecules including CagA to be translocated into the host cell where subversion of host cell signalling occurs. HtrA is secreted by the bacteria and cleaves intracellular cell junctions allowing bacteria to gain access to the basolateral cell surfaces providing further access to receptors for the *cag*T4SS pili and VacA. Cellular events that occur following CagA translocation into the host cell include CagA phosphorylation, inactivation of Src kinase, cytoskeletal rearrangements, further disruption of cell junctions, increased cell mobility, cell proliferation, and induction of proinflammatory cytokines and chemokines.

While most *H. pylori* organisms are found in the mucus layer in the stomach, interaction of the organisms with the underlying epithelial cells is necessary for pathology to occur. An early study provided clear evidence that the interaction of *H. pylori* with gastric epithelial cells was associated with pathology [165]. Histological staining and transmission electron microscopy were used to assess pathology and to visualize *H. pylori* in human gastric biopsy specimens from 40 infected individuals. Bacteria were seen attached to surface epithelial cells, between cells or accumulated at the base of gastric pits. Some organisms formed adherence pedestals and others formed indentations at the sites of attachment. There was a direct correlation between the number of organisms adherent to the epithelial cells and epithelial degeneration, microvilli destruction, and mucin depletion. Ruthenium red staining showed a fuzzy layer on the surface of the bacteria including at the site of attachment indicating a role for glycosylated molecules in adhesion. Over the last three decades the interaction of *H. pylori* with the gastric epithelium has been extensively investigated, and several bacterial virulence factors have been identified that enable *H. pylori* to signal to the host cell, and cause chronic infection. A summary of how some of the virulence factors of *H. pylori* mediate inflammation and pathology is shown in Figure 3.

#### CagA and the cagPAI

Probably the best characterized virulence factor of *H. pylori* is the CagA protein and the *cag* pathogenicity island (PAI). The *cag*PAI contains approximately 30 genes and encodes for a type 4 secretion system (T4SS) that acts to translocate the CagA protein and other effector molecules into the cytoplasm of host cells, resulting in subversion of host cell signalling. CagA was first identified as an immunodominant antigen, and seropositivity in infected humans was significantly associated with polymorph infiltration, epithelial degeneration, and peptic ulceration [166]. It is now also strongly associated with the development of gastric cancer [167]. Synthesis of needle like pili by the *H. pylori* T4SS is induced upon contact of the bacteria with epithelial cells and can be visualised using electron microscopy [168]. CagL is a highly conserved protein found at the tip of the pilus and can bind to and activate α5β1 integrin via an RGD motif, thereby triggering delivery of CagA to the host cell and subsequent activation of tyrosine kinase Src, and focal adhesion kinase [169]. In addition, specific binding of CagY, a pilus surface protein, and the CagA protein to β1 integrin receptor was shown to occur and subsequent CagA translocation was dependent on a conformational change of the integrin from the active to the inactive form [170]. A further study using surface plasmon resonance and purified proteins demonstrated that CagI, CagA, CagL and CagY interact with the ectodomain of α5β1 integrin. Integrin activation increased the interaction with CagA, CagL, and CagY, and CagI interaction was dependent on integrin activation. This study also demonstrated novel interactions between CagA and CagL, and between CagY and CagI. The reason why CagA might interact with surface exposed proteins on the pilus is unknown, but it is speculated that it may act to stabilize the pilus in some way and may explain why more CagA is produced by the bacteria than is translocated into the host cell [171].

Translocation of CagA into the host cell is dependent on the interaction of outer membrane protein HopQ with carcinoembryonic antigen related cell adhesion molecules (CECAMs). CECAM 1, 5 and 6 were shown to be expressed at low levels in healthy gastric tissue but expression was highly induced upon infection. *H. pylori* has been shown to bind to CECAMS 1, 3, 5, and 6 [172,173]. Deletion of CECAMs 1, 5 and 6 in Kato III cells abolished CagA translocation almost completely although adherence was only reduced by about 50% [174]. Expression of CECAM 1 or 5 in intestinal cell line AZ-521 that does not naturally express CECAMs, enabled CagA translocation to occur [175] indicating that, depending on the cell model, different CECAMs may be involved. CECAM expression on neutrophils has been shown to allow HopQ mediated adherence and CagA translocation to occur, leading to enhanced expression of the proinflammatory chemokine Mip1α. Expression of human CECAM in a murine model of infection allowed for the extended survival of *H. pylori* within the phagosome. During chronic infection of humanized mice the expression of CECAMs 1 and 6 on neutrophils was decreased, suggesting that the host may try to prevent CagA translocation to limit gastric pathology [176].

CagA once translocated into the host cell can be tyrosine phosphorylated by host cell kinases Src [177,178] and Abl [179]and this starts a series of events that result in actin cytoskeletal remodelling [178,180], disruption of epithelial junctions [181], and production of an inflammatory response [182]. Tyrosine phosphorylation of CagA occurs at EPIYA motifs the number of which can vary between isolates [183]. Four phosphorylation EPIYA motifs EPIYA-A, B, C and D exist with motifs A, B and C found predominantly in Western isolates, and A, B and D in East Asian isolates. Src acts to phosphorylate EPIYA C and D motifs, while Abl can phosphorylate all four motifs [184]. Once CagA is phosphorylated by Src kinase it can then inactivate Src, leading to inhibition of CagA phosphorylation in a negative feedback loop. Inactivation of Src also results in tyrosine dephosphorylation of actin binding proteins and of Src targets such as cortactin, ezrin and vinculin. These events culminate in cytoskeletal rearrangements and trigger cell elongation and mobility [185]. Late in the infection when Src is inactivated, CagA phosphorylation can be maintained by Abl kinase which promotes cell mobility [186].

CagA can also induce events in the host cell that are phosphorylation independent. Non-phosphorylated CagA interacts with adherens junction protein E-cadherin and impairs the interaction between E-cadherin and β-catenin resulting in accumulation of β-catenin in the cytoplasm, and in the nucleus of the cell [187]. Non-phosphorylated CagA has been shown to interact with the hepatocyte growth factor Met, and this interaction contributed to cellular proliferation and inflammation via downstream PI3K/Akt signalling, NFkb signalling and β-catenin activation [188]. Non-phosphorylated CagA has also been shown to decrease autophagy and increase inflammation regulated by activation of the c-Met-Pi3K/Akt-mTOR signalling pathway in *H. pylori* infected gastric biopsies and in infected gastric cells [189]. Thus, translocated CagA that is not phosphorylated or is dephosphorylated, can still contribute to events in the host cell that will promote carcinogenesis. For detailed reviews of CagA and T4SS mediated events see earlier reviews [190,191].

#### CagL and CagY

Recombinant CagL stimulation of IL-8 expression in gastric epithelial cells was dependent on the interaction of CagL with α5β1 integrin but independent of CagA translocation, and involved mitogen activated protein kinase and NFkB activation [192]. The CagL adhesin protein has also been shown to trigger production of the inflammatory cytokines IL-6 and IL-8 in primary human endothelial cells. This event was independent of CagA translocation and of the interaction of CagL with α5β1 integrin, but dependent on epidermal growth factor receptor activation [193]. Recently it has been shown that T4SS proteins CagL [102] and CagY [103] can both activate TLR5. CagL was shown to have a D1-like domain that can mediate attachment of CagL to TLR5 positive epithelial cells and initiate TLR5 activation. Infection of *Tlr5* knockout and wild type mice indicated that TLR5 plays a role in controlling *H. pylori* infection [102]. Likewise, the CagY protein was shown to have TLR5 interacting sites, and be capable of activating TLR5, and initiating intracellular signal transduction. Interestingly, TLR5 expression was found to be low in healthy gastric tissue but was strongly upregulated upon infection and the level of expression correlated with the severity of disease [103]. Interaction of T4SS surface proteins with TLR5 may be an important mechanism used by *H. pylori* to control the innate immune response, as unlike flagellin expression, expression of the T4SS can be more easily modulated by the bacteria.

#### Peptidoglycan

Intracellular delivery of a *H. pylori* peptidoglycan motif was shown to be dependent on the presence of the *cag*T4SS. Once translocated this motif was recognised by the intracytoplasmic sensor molecule Nod-1. Nod-1 deficient mice were shown to be more susceptible than WT mice to infection with T4SS positive bacteria, indicating that Nod-1 sensing of the translocated peptidoglycan played a role in the host sensing of this pathogen, and in initiation of innate host defence systems [194]. Infection of gastric epithelial cells with T4SS positive bacteria resulted in secretion of human beta defensin 2 which can kill *H. pylori*, but this effect was abolished when Nod-1 was knocked down in the cells [195]. Recently it has been shown that induction of IL-18 by gastric epithelial cells is induced upon infection with *H. pylori* and is dependent on Nod-1 recognition of peptidoglycan. IL-18 production was shown to be important for limiting gastric pathology during chronic infection, and infection of gastric organoids derived from *Nod1+/+* and *Nod1-/-* mice showed higher levels of cell proliferation, and apoptosis occurred in organoids from the Nod-1 deficient mice [196]. These results indicate that recognition of *H. pylori* peptidoglycan by Nod-1 is important for maintenance of epithelial cell homoeostasis upon infection.

#### Chromosomal DNA

TLR9 reacts with hypo-methylated CpG DNA motifs found in bacterial genomes. Using a HEK293 reporter assay *H. pylori* genomic DNA was shown to activate TLR9 as was WT *cag+ H. pylori* strains. Infection assays with *H. pylori* WT strains and knockdown mutants where the DNA was labelled with BRDU showed BRDU labelled DNA in cells infected with WT *cag+* strains but not in *cagE* or *cagY* mutant strains indicating that a functional *cag*T4SS was required for translocation of the DNA into the host cells [197]. Interestingly, *H. pylori* isolates from regions with a high rate of gastric cancer activated TLR9 to higher levels than isolates from regions with a low rate of gastric cancer and the levels of TLR9 expression in gastric tissue of individuals from high-risk regions were significantly higher than in individuals from low-risk regions [197]. In another example of how the *cag*T4SS is used by *H. pylori* to reduce the intensity of the immune response, infection studies in *Tlr9-/-* and *1L17A-/-* mice demonstrated that activation of TLR9 by *H. pylori* results in an anti-inflammatory phenotype with a reduction in 1 L–17 mediated responses [198].

#### LPS

The LPS of *H. pylori* is distinctive due to its low inflammatory properties compared to the LPS of other enteric bacteria [199,200] which is thought to be due to the presence of tetraacyl lipid A with long chain fatty acids of 16 to 18 carbons in length and an unusual phosphorylation pattern [201,202]. *H. pylori* LPS activated TLR2 but not TLR4, which is usually activated by Gram negative LPS, in HEK-293 cells transfected with TLR2 and TLR4 plasmids [203,204]. Recognition of TLR2 by *H. pylori* results in NFkb activation, production of IL-8 [204], disruption of adherens junctions in epithelial cells [205], increased TLR2 expression, and increased expression of claudins 4, 6, 7, and 9, associated with cellular invasiveness and metastatic potential [206]. In contrast *H. pylori* LPS was found to signal through TLR4 and MyD88 and not TLR2 to induce IL-1β secretion when tested using murine bone marrow derived dendritic cells (DCs) [207].

The intracellular presence in *H. pylori* infected cells of heptose-1,7-bisphosphate (HBP), an intermediate metabolite of the LPS inner heptose core, has been shown to be dependent on the *cag*T4SS and play a role in induction of proinflammatory signalling and IL-8 secretion in gastric epithelial cells. Mutants lacking genes required for synthesis of HBP exhibited reduced IL-8 induction, and *cag*T4SS-dependent cellular signalling. The cellular adaptor TIFA was shown to be vital in the *cagPAI*- and HBP dependent responses induced by *H. pylori* in different cell types [208,209]. HBP translocation drove robust NFkB dependent inflammation early in infection and preceded Nod1 activation. Subsequent CagA translocation contributed further to the NFkB driven response [208]. Recently A heptose-monophosphate variant was also identified as a novel proinflammatory metabolite and regulation of heptose production was shown to be strain specific with the cag pathogenicity island, interaction with human cells, and the carbon starvation regulator A all found to play a role [210].

Structures that mimic Lewis blood group antigens are found on the O antigen of *H. pylori* LPS [211,212]. Lewis structures found on LPS include the type I structures, Lewis a and Lewis b and type 2 structures Lewis x and Lewis y. Expression of type 2 structures is more common than expression of type 1 is [213,214]. Molecular mimicry of these antigens by *H. pylori* LPS is thought to play a role in immune evasion [215,216] and induction of autoantibodies [217]. Interestingly molecular mimicry of blood group antigens has also been found in the LPS of *H. mustelae* [218,219], a species of *Helicobacter* that infects ferrets who also develop chronic gastritis and, peptic ulceration [220], and produce autoantibodies in response to infection [221]. Lewis x structures have been suggested to mediate adherence of *H. pylori* to galectin 3, expression of which is upregulated in gastric tissue upon infection [222,223].

The core oligosaccharide segment of *H. pylori* LPS has been identified as a binding partner for TFF1 [224,225], a member of the trefoil peptide family of proteins that can interact with *H. pylori* [226]. TFF1 is found in mucus and is co-expressed by goblet cells with the gastric mucin MUC5AC [125], the expression of which co-localises with *H. pylori* [127]. The interaction of *H. pylori* with TFF1 is modulated by pH, with optimum binding occurring at near neutral pH [225]. The interaction of *H. pylori* with TFFI has also been shown to promote aggregation and decrease the speed with which *H. pylori* can traverse gastric mucin [227]. It has been suggested that the interaction between *H. pylori* LPS and TFF1 may serve to locate the organism in gastric mucus and restrict its interaction with epithelial cells, thus limiting subsequent host signalling events that promote inflammation and thereby promoting persistence [227,228].

Interestingly it has recently been reported that adhesins of *H. pylori*, BabA, BabB and AlpA and AlpB are glycosylated and the LPS biosynthesis machinery plays a key role in glycosylation of these proteins. Mutations in LPS biosynthesis genes reduced protein glycosylation and caused a significant reduction in bacterial adhesion to AGS, gastric epithelial cells [229]. This opens the possibility of using LPS biosynthesis enzymes as therapeutic targets for *H. pylori* infection.

#### HtrA

High-temperature requirement A (HtrA) is a protein chaperone and serine protease found in prokaryotic and in eukaryotic cells. HtrA is secreted by *H. pylori* [230] and can cleave the tumour suppressor protein E-cadherin found in adherens junctions [231], tight junction proteins occludin and claudin-8 [232], and desmoglein found in desmosomes [233]. Thus *H. pylori* HtrA can disrupt each of the different types of intercellular epithelial junctions that function to promote and maintain the barrier function and integrity of epithelial sheets. Disruption of epithelial cell junctions allows for paracellular transmigration of the bacteria to the basal side of the epithelium where integrin molecules are normally expressed. Interestingly the T4SS pilus is predominantly expressed when bacteria interact with the basal and basolateral side of the cell membrane [232], thus, secretion of HtrA, and cleavage of cell junctional proteins provides a mechanism for *H. pylori* to traverse the gastric epithelium, thereby gaining access to integrin receptors, allowing for T4SS pilus formation, and translocation of the CagA protein and other effector molecules into the host cell. Furthermore, HtrA expression promotes localisation of *H. pylori* with epithelial tight junctions [234]. In a study of 100 global strains, it was only possible to delete or mutate *htrA i*n one strain, strain N6. Chromosomal sequencing of the mutated and WT strains showed that mutation of *htrA* is associated with mutations in the ATPase SecA, a component of the Sec machinery used to translocate proteins across the cytoplasmic membrane [235].

Genome wide RNA seq analysis of polarized MKN28 gastric cells infected by WT strain N6 and a *htrA* knockout strain revealed that infection with *htrA* negative bacteria resulted in increased apoptosis and reduced CagA expression [234]. A HtrA single nucleotide polymorphism (SNP) has been identified (position serine/leucine 171) and the 171 L type HtrA promoted HtrA trimer formation. HtrA trimers were associated with enhanced cleavage of junctional proteins, translocation of CagA, NFkB mediated inflammation and cell proliferation through nuclear accumulation of β-catenin and induction of host DNA double strand breaks [236]. Together these events act to trigger malignant changes in infected tissue.

#### VacA

VacA is a 94 kDa secreted toxin [237] that induces vacuolization of epithelial cells *in vitro* [238–240] and mucosal ulceration in mice [237] and is associated with peptic ulceration and gastric cancer in humans [238,241,242]. The toxin inserts into host cell membranes where it forms chloride sensitive channels. VacA causes a range of effects on cells including the induction of apoptosis [243], alterations in antigen presentation [244] and inhibition of T and B cell activation [245–247]. VacA also plays an essential role in induction of a tolerogenic phenotype in DCs that in turn acts to promote regulatory T cell differentiation, which facilitates persistent infection [248,249]. Autophagy is induced in *H. pylori* infected cells in a VacA dependent manner but prolonged exposure of gastric cells to VacA disrupts autophagy [250] and lysosomal signalling pathways generating an intracellular space where *H. pylori* can be protected from antibiotics. VacA has been shown to target the lysosomal calcium channel TRPML1 and disrupt endolysosomal trafficking and this effect could be reversed by treatment with a small molecule agonist directed against TRPML1, suggesting that TRPML1 is a potential therapeutic target for chronic *H. pylori* infection [251].

Sequence variation exists in each of several regions present in *vacA*, including the s region, a 5’ region encoding the signal sequence and the amino terminus of the protein, the i or intermediate region and the m or mid region [238,242]. The polymorphism that exists in *vacA* results in both active and inactive forms of the toxin produced by different strains. The s1 allelic form produces toxigenic VacA, while s2 types possess a 12-residue hydrophilic extension in the N terminus and are generally considered to be less or non-toxigenic [252]. Sequence variation in the N terminal part of the m region has been shown to influence VacA toxicity [253]. A chimeric m2/m1 VacA protein was less toxigenic than an m1 VacA protein when tested against gastric and duodenal cells and interestingly both m1 and m2/m1 forms of VacA bound at a higher level to the basolateral surface of polarized gastric organoid cells than to the apical surface [254]. This finding further highlights the role of HtrA in promoting infection as disruption of epithelial junctions would allow VacA access to receptors which may be present in higher numbers on the basolateral surface.

Other regions of *vacA* where genetic diversity exists are the d (deletion) and c (central) regions. The d region occurs between the m and i regions and the d1 and d2 regions differ on the presence or absence of up to 81 bps [255]. In western isolates the presence of the d1 region was significantly associated with neutrophil infiltration and development of gastric atrophy [255]. Genotyping of isolates from Iran revealed a strong correlation between the d1 region and the development of gastric cancer and with peptic ulcer disease [256]. The c region is defined by the presence (c1) or absence (c2) of a 15 bp nucleotide sequence. Studies from Iran have linked the presence of the c1 allele to an enhanced risk of gastric cancer [257,258] as did a study from China [259].

A study designed to examine the effect of different forms of VacA on inflammation and metaplasia in a mouse model of infection found strains producing active VacA forms induced more extensive metaplasia and inflammation than strains producing less active s2/i2 VacA. Furthermore, examination of infected human gastric biopsy specimens revealed a strong association between the presence of the *vacA* i1 allele and intestinal metaplasia, with minimal intestinal metaplasia detected in biopsies with i2-type strains [260]. In experimental infection of mice, a null mutation of *vacA* compromises *H. pylori* in its ability to establish initial infection and the presence of VacA reduces the infectious dose [261], suggesting that *vacA* confers a fitness advantage on *H. pylori* and likely explains why all strains of *H. pylori* possess *vacA*. Strong evidence of a positive role for the minimally active s2/i2 VacA form is that bacteria producing this form of VacA colonize mice more efficiently than *vacA* null mutants or strains expressing more active forms of VacA [260].

#### Gamma glutamyl transpeptidase

Another secreted protein of *H. pylori* that has been shown to play an essential role in the establishment of and the persistence of infection is γ-glutamyl transpeptidase (GGT) [249,262,263]. GGT is an enzyme found in the periplasm and is also secreted from the bacteria, that catalyses the transpeptidation and hydrolysis of the γ-glutamyl group of glutathione and related compounds. The physiological function of GGT in *H. pylori* is to enable the bacteria to scavenge and use extracellular glutamine and glutathione as a source of glutamate. This deprives host cells of these two nutrients and may render them susceptible to damage by the organism [264]. However, supplementation of infected cells with reduced glutathione resulted in increased cell death, and glutathione degradation products were shown to play a direct role in cell death by necrosis and apoptosis [265]. GGT promotes the degradation of survivin [266], a protein that plays a role in inhibition of apoptosis by blocking caspase activation. GGT inhibits T cell activation and proliferation [267,268] and cytokine expression via glutamine deprivation in the extracellular environment [268]. In a murine model of infection, GGT could induce a tolerogenic phenotype in DCs independently of the effect of VacA [249]. Glutamate produced by the enzymatic activity of *H. pylori* GGT is able to tolerize human DCs by inhibiting cAMP signalling and dampening IL-6 secretion, which promotes the expansion of naïve T cells and production of T reg cells [269].

#### Neutrophil activating protein (NapA)

NapA is a 15 kDa iron binding protein [270]. Expression is upregulated in late log phase during growth *in vitro* [271]. It plays a role in regulation of the *H. pylori* immune response. The protein can activate neutrophils and monocytes and is a powerful stimulant for production of reactive oxygen radicals [270]. *H. pylori* NapA upregulates the expression of β2 integrins on leukocytes promoting neutrophil adhesion [272]. NapA induces a moderate inflammatory reaction resulting in disruption of epithelial tight junctions and basal membranes, allowing the protein access to underlying tissue, where it is a potent stimulator of mast cells inducing the release of IL−6 [273]. The protein acts as an agonist for TLR2 and promotes a Th1 type immune response. NapA induced T cell lines showed an increase in IFN-γ producing cells and a decrease in IL-4 production. Neutrophils and monocytes induced by NapA showed IL-12 and IL-23 expression [274].

NapA plays a role in oxidative stress resistance and *napA* mutants are more sensitive to oxygen than wild type strains are [275]. In the presence of iron NapA can bind DNA thereby protecting the DNA against iron mediated oxidative stress damage [276]. A recent study has shown that H_2_O_2_ - induced oxidative stress promoted *H. pylori* biofilm formation. Stress regulation protein, SpoT, upregulated expression of NapA and oxidative stress-induced biofilm formation. Furthermore, treatment of biofilms with *N*-acetylcysteine or vitamin C decreased *H. pylori* biofilms and downregulated *napA* expression. Biofilms induced by oxidative stress were more resistant to antibiotics than those induced by nutrient starvation [277]. *H. pylori* can form biofilms on the gastric surface [278] and in gastric glands [58]. Targeting of NapA may be a potential mechanism for prevention of drug resistance in *H. pylori* and is already considered as a strong candidate for inclusion in a *H. pylori* vaccine [279].

#### IceA

*H. pylori* adherence to gastric epithelial cells stimulates transcription of the *iceA* gene (induced by contact with epithelial cells), for which there are two different alleles *iceA1* and *iceA2*. The presence of *iceA1* in clinical isolates was highly correlated with peptic ulceration and increased IL-8 concentrations [280]. However, a subsequent study looking at isolates from patients with different symptoms and from different geographical regions found that neither *iceA* nor combinations of *iceA* with *cagA* and *vacA* genotypes were useful for predicting clinical outcome [281]. There exists a range of studies from different geographical regions of the world, with some finding an association between *iceA* and disease outcome [282–288]and others finding no association [289–292]. These conflicting results are likely due to studies with insufficient numbers of isolates. Other factors that need to be considered include the presence of multiple bacterial virulence determinants, isolates with different genotypes in the same host, host genetic polymorphisms which predispose individuals to certain disease outcomes, and environmental factors that prevail in different geographical locations [293].

#### DupA

The *dupA* (duodenal ulcer promoting) gene of *H. pylori* was identified in a study that examined 500 *H. pylori* strains from individuals in East Asia and South America with gastritis, duodenal ulcer, gastric ulcer, or gastric cancer. The presence of *dupA* was associated with neutrophil infiltration, high levels of IL-8, and protection against gastric atrophy, and gastric cancer. Of note however, more than 50% of duodenal ulcer isolates did not possess *dupA* [294], highlighting the need to consider multiple factors when attempting to predict disease risk. Another study looking at 482 strains from children and adults in Brazil found that *dupA* was highly prevalent but that there was no association with disease [295]. However, a subsequent study from the same group found 2 mutations in the *dupA* gene that acted as stop codons, and the presence of the *dupA* gene without any mutations was associated with increased antral inflammation, increased IL-8 levels in the gastric mucosa, and decreased gastric atrophy [296]. A recent study looked at the microbial diversity in gastric mucosal biopsies from *dupA+* and *dupA- H. pylori* infected individuals. Although infection with either *dupA+* or *dupA- H. pylori* reduced microbial diversity, infection with *dupA+ H. pylori* caused fewer changes to the microbiome, and microbes that produce beneficial metabolites were more likely to be maintained. This resulted in less carcinogenic metabolites in the tissue [297]. These findings merit further investigation using a larger number of clinical samples from different geographical locations and studies using cellular and animal models.

#### OipA

OipA (outer inflammatory protein) is an outer membrane protein, also referred to as HopH, that enhances IL-8 production when *H. pylori* isolates are cultured with gastric epithelial cells. Slipped strand mispairing based on the number of CT dinucleotide repeats in the 5’ region of *oipA* result in a frame shift mutation, and strains with these mutations do not induce IL-8 production [298]. A functional *oipA* was found to be related to clinical symptoms, *H. pylori* density, and gastric inflammation [299], and has been linked to expression of other virulence markers, namely the *cag*PAI, *vacA* and *babA2* genotypes [299,300]. Dossumbekova *et.* *al.* did not observe enhanced IL-8 induction in strains with a functional *oipA* genotype, but *oipA* mutants exhibited reduced adherence to gastric epithelial cells [300]. It has been suggested that the strong association observed between a functional *oipA* and *cag*PAI may indicate that *oipA* contributes to the fitness of *cag*PAI positive strains [301]. Horridge *et.* *al.* found that a functional *oipA* was necessary but not sufficient for IL-8 induction and was required for efficient CagA translocation. They also observed enhanced upregulation of *oipA* mRNA in bacteria adherent to gastric epithelial cells compared to non-adherent bacteria [302]. Bacteria with an *oipA* off or non-functional status or deletion of *oipA* caused higher levels of apoptosis and cell cycle arrest in gastric epithelial cells than wild type isolates with a functional *oipA* status did. Furthermore, deletion of *oipA* resulted in increased production of VacA protein and vacuolating activity [303]. A recent molecular epidemiological study of 322 isolates from USA and Korea found that most Korean isolates possessed 2 copies of *oipA*, and possessed 3 or less CT repeats, while all USA isolates contained only one copy of *oipA* with five or more CT repeats. All Korean isolates possessed at least one functional phase variant but only 56% of USA isolates did. The duplication of *oipA* with 3 or less CT repeats ensures continued expression of *oipA* in the Korean isolates suggesting there is selective pressure in this population for a functional *oipA* [304].

#### T4SS comB

*H. pylori* is a naturally competent organism but lacks type 4 pili or pilin like proteins commonly found in other competent bacteria. The *comB* locus in *H. pylori* consisting of 4 tandemly arranged genes *orf2* and *comB*8, *comB*9 and *comB*10 was identified. Tn mutagenesis studies where the *comB* genes were knocked out resulted in phenotypes where natural transformation was either severely or completely inhibited [305]. Subsequently it was shown that all ComB proteins and a 37 AA ORF2 peptide displayed significant sequence and structural homology to a T4SS apparatus. Complementation of a *H. pylori comB* deletion mutant showed that *comB*8, *comB*9 and *comB*10 are all essential for natural transformation to occur [306]. Thus *H. pylori* possesses a T4SS for the uptake of DNA by natural transformation.

### Treatment and eradication of *H. pylori* infection

Since the discovery of *H. pylori* and its association with gastritis, combined treatments with acid suppressors and multiple antibiotics have been used effectively to eradicate the infection [307,308] The acid suppressors are required to increase the pH around the bacteria allowing them to be more metabolically active, and thus more susceptible to the antibiotics used [309]. The most widespread approach historically has been the use of two antibiotics with a proton pump inhibitor (PPI) termed triple therapy with the most used antibiotics being clarithromycin and amoxycillin or metronidazole. Initially triple therapy was very effective with complete clearance of infection in most cases [310,311]. However, over time an increased number of treatment failures occurred, and second line more advanced therapies were developed [311]. These included quadruple therapies which involved the addition of bismuth to the proton pump inhibitor, and two antibiotics that make up traditional triple therapy [312]. Bismuth, like the proton pump inhibitors, helps to render *H. pylori* more susceptible to antibiotics by making the organism more metabolically active and increasing its intracellular pH. This quadruple therapy approach has been shown to have increased effectiveness over triple therapy with eradication in 90% of cases [313]. Other approaches to increase eradication rates have involved the use of new classes of acid inhibitors to further increase the potency of the antibiotics that are used in these therapies [314]. Studies with vonoprazan, a potassium competitive acid blocker, in dual or triple therapy have shown eradication levels greater than those observed with traditional triple therapy regimes [315,316].

The need for the development of new treatment strategies is driven in part by a stark increase in antibiotic resistance in *H. pylori* strains world-wide. This increase in resistance is emerging to many of the antibiotics currently being used in first-line triple therapy treatment. They include metronidazole and clarithromycin where primary and secondary resistance was recently reported to be greater than 15% in all regions tested in a global study and significantly higher in some regions [317]. In 2017 the World Health Organization identified clarithromycin resistant *H. pylori* as one of twelve priority pathogens which require the identification of new antibiotics. It has been postulated that the misuse of clarithromycin has led to this increase and that careful stewardship is required to identify which antibiotics should be used in treating *H. pylori* infection in different geographical regions [318]. As resistance levels to key antibiotics rise it will be critical to ensure that the resistance profile of infecting strains is considered when deciding which combination of antibiotics is used in treatment [319].

An alternative option to counteract the rise in antibiotic resistance in *H. pylori* worldwide would be to develop an effective vaccine to protect children against infection. Although much is known about the immune response during infection in adults it has been shown that the immune response in children is very different as children display significantly lower levels of inflammation [320]. This may pose a challenge in eliciting an effective immune response in this cohort. Despite this challenge many vaccine candidates have been identified, and promising results have been observed in mouse models using whole cell [321,322], vector delivered [323,324], sub-unit [325]and DNA [326] based vaccine approaches. Early studies revealed that vaccines could be used to therapeutically treat pre-colonised mice [321,322]. However, despite the success in reducing or eradicating *H. pylori* infection in mouse models there has been less success when vaccines have progressed to human trials. Several vaccines have been tested in humans with some eliciting effective immune responses [327,328], but none to date showing signs of effective eradication of infection. However, in 2015 it was reported that an oral recombinant based vaccine administered to children in China resulted in a significant reduction in *H. pylori* infection in a double blind, placebo-based study [329]. A follow-up over a longer time will tell whether this promising vaccine candidate could be an effective tool against *H. pylori* associated disease.

### Biofilm formation by *H. pylori*

*H. pylori* have been found in biofilms formed in the gastric glands in vivo [58]. Biofilms are structures which encase microorganisms in extracellular material. They can play an important role in enabling survival in different environments, especially in the presence of antibiotics, as antibiotics often are unable to penetrate biofilm material. Biofilms form at the air liquid interface when *H. pylori* is grown in batch culture on glass slides [330]. *H. pylori* biofilms contain outer membrane vesicles [331,332], proteomannans [333], and eDNA [334]. Expression of proteins such as AlpB and NapA has been shown to play a role in mediating adhesion of *H. pylori* organisms in biofilms [277,335]. Expression of *homB*, encoding for outer membrane protein HomB, is increased in *arsRS* mutants which display enhanced biofilm formation. Loss of *homB* from hyper-biofilm forming strains resulted in a biofilm phenotype that mimicked wild-type biofilm indicating that *homB* plays a role in mediation of hyper-biofilm formation [336]. A high level of expression of efflux pumps have been found in biofilm cells and biofilm formation has also been shown to be associated with the presence of point mutations that mediate resistance to clarithromycin [337]. Exposure of *H. pylori* to calprotectin, a calcium and zinc binding protein associated with inflammation, results in altered lipid A modification, increased cell surface hydrophobicity and increased biofilm formation [338]. Not surprisingly strategies to disrupt biofilm formation are increasingly being studied as a way to overcome antibiotic resistance in *H. pylori*. For a comprehensive review of *H. pylori* biofilm formation and its role in antibiotic resistance and immune evasion see reference [339].

### Diagnostic tests and the need for antibiotic sensitivity testing

*H. pylori* is a very slow growing organism and is fastidious in its growth requirements. It is a microaerophilic bacterium requiring blood or serum for growth and takes 5–7 days to culture from gastric biopsy specimens [1]. Histology can be used to visualise the organism directly in gastric tissue [340]. Both culture and histology are time consuming, but the discovery of the particularly potent urease enzyme led to the ability to detect the organism using the rapid urease test. Immersion of an infected biopsy specimen in a solution containing urea and phenol red results in a colour change as the pH rises due to the conversion of urea to ammonia [341]. This led in turn to the development of a non-invasive based test, the urea breath test involving the ingestion of a solution containing ^14^C or ^13^C radio-labelled urea and detection of radio-labelled exhaled CO_2_ by mass spectrometry [342,343]. The urea breath tests were shown to be both safe and accurate in patients over the age of two [344] and although costly, they are importantly non-invasive. Other non-invasive tests include serology or stool antigen tests. Serology is a useful indicator of infection but cannot differentiate between a current or previous infection making it primarily useful for screening or epidemiological studies [345,346]. The stool antigen test is more effective at identifying an active infection, but the quality and storage of samples are critical to maintain high levels of sensitivity and specificity [347,348].

While non-invasive tests are convenient and are used increasingly in the clinic and in epidemiological studies, they only detect the organism and do not allow for the diagnosis of pathology such as duodenal ulceration or precancerous changes that may occur in the stomach. In addition, given the previously mentioned rise in resistance to antibiotics, and increased rate of treatment failure in the clinics, antibiotic sensitivity testing is becoming essential for effective treatment strategies [349]. Antibiotic sensitivity testing can be carried out phenotypically by direct culture from biopsies or alternatively by direct PCR based genotyping on biopsies or stool samples [350,351]. Screening of strains for their antibiotic susceptibility profile prior to treatment is the best hope to combat the rising levels of antibiotic resistance observed in this important human pathogen.

## Concluding remarks

A search of Pubmed revealed for us that since *H. pylori* was first cultured in 1984 and its subsequent identification as a pathogen responsible for causing serious gastric pathology there have been close to 60 other *Helicobacter* species, or potential species identified to date. These have been found in different animal species ranging from rodents to dolphins to birds, and in humans. Some of these are gastric *Helicobacters* while others are enterohepatic. However, the species most studied to date is *H. pylori* which has only ever been found in primates except for one finding of infection in a colony of cats [352]. The interplay of bacterial host and environmental factors all contribute to determine the pathology caused by *H. pylori* and make prediction of the outcome of infection in different individuals particularly challenging. A recent study on *H. pylori* genetic diversity in different environmental niches looked at 10 isolates from each of 16 infected individuals. Results revealed that the bacteria evolve differently in different locations in the stomach and that chemotaxis, regulatory functions, and outer membrane proteins play a special role in adaptation of the organism to its environment. This study also identified antibiotic use as an important factor in determination of the *H. pylori* population structure [353].

Particularly important when studying *H. pylori* pathogenesis is the choice of animal models. The earliest and probably the best animal models of *H. pylori* were infection of gnotobiotic piglets [354] and infection of non-human primates [355]. However, the expense, the technical difficulties and the ethics involved make studies with these large animals prohibitive for most investigators. Other animal models used in the past were *H. felis* infection of mice [356] and *H. mustelae* infection of ferrets [357], but both models had their limitations. *H. pylori* infection of the mouse is the most widely used model of infection to date but the extent of inflammation that develops in mice is usually milder than that seen in humans [358]. Probably the best small animal model developed, especially for studies on gastric cancer, has been infection of the Mongolian gerbil. Gerbils develop a more vigorous inflammatory response than that seen in mice and can develop ulcers [359], and gastric cancer [360] following infection. However, one of the great advantages of working with *H. pylori* is the availability of naturally infected tissue to study the effect of the bacteria in the human stomach and for validation of experimental findings. In addition, the use of human tissue for gastric organoid culture is a significant *ex-vivo* model system that has been and is being used extensively for studies on *H. pylori* [361].

Important questions that we cannot answer yet about *H. pylori* is how the organism is transmitted from person to person, how exactly it manages to establish chronic infection, why the majority of people have asymptomatic infection while others develop serious disease, is it possible to develop a vaccine for humans who cannot eradicate this chronic infection naturally and if so, who should receive such a vaccine. The importance of future studies on *H. pylori* is underlined by the problem of antimicrobial resistance and the need for alternative therapies. In a recent study treatment of *H. pylori* infected mice with dimethyloxalyglycine (DMOG), a prolyl hydroxylase inhibitor that stabilises HIF-1α, resulted in attenuation of *cag*-mediated virulence and suppression of host proinflammatory responses [362]. The development of such approaches could lead to new therapeutic approaches in humans and limit the development of gastric cancer.

## Data Availability

Data sharing is not applicable to this article as no new data were created or analysed in this study.
